# The impact of time from injury to surgery in functional recovery of traumatic acute subdural hematoma

**DOI:** 10.1186/s12883-020-01810-4

**Published:** 2020-06-04

**Authors:** Shih-Han Chen, Jui-Ming Sun, Wen-Kuei Fang

**Affiliations:** Neurosurgical Department, Ditmanson Medical Foundation Chia-Yi Christian Hospital, No. 539, Zhongxiao Rd, Chia-Yi City, Taiwan 60002

**Keywords:** Time from injury to surgery, Traumatic acute subdural hematoma, Surgical outcomes of TASDH, ROC curve

## Abstract

**Background:**

The time from injury to surgery (TIS) is critical in the functional recovery of individuals with traumatic acute subdural hematoma (TASDH). However, only few studies have confirmed such notion.

**Methods:**

The data of TASDH patients who were surgically treated in Chia-Yi Christian Hospital between January 2008 and December 2015 were collected. The significance of variables, including age, sex, traumatic mechanism, coma scale, midline shift on brain computed tomography (CT) scan, and TIS, in functional recovery was assessed using the student’s *t*-test, Mann-Whitney U test, chi-square test, univariate and multivariate models, and receiver operating characteristic (ROC) curve.

**Results:**

A total of 37 patients achieved functional recovery (outcome scale score of 4 or 5) and 33 patients had poor recovery (outcome scale score of 1–3) after at least 1 year of follow-up. No significant difference was observed in terms of age, sex, coma scale score, traumatic mechanism, or midline shift on brain CT scan between the functional and poor recovery groups. TIS was found to be significantly shorter in the functional recovery group than in the poor recovery group (145.5 ± 27.0 vs. 181.9 ± 54.5 min, *P*-value = 0.002). TIS was a significant factor for functional outcomes in the univariate and multivariate regression models. The analysis of TIS with the ROC curve between these two groups showed that the threshold time for functional recovery in comatose patients and those with TASDH who were surgically treated was 2 h and 57.5 min.

**Conclusions:**

TIS is an important factor l for the functional recovery of comatose TASDH patients who underwent surgery.

## Background

Traumatic acute subdural hematoma (TASDH) is one of the most devastating types of traumatic brain injury (TBI), with a mortality rate ranging from 30 to 70% [1–4]. An emergent operation is considered if a patient is in coma or meets the surgical indication for TASDH. In 1981, Seelig et al. (1981) have reported that the mortality rate of TASDH can be reduced from 90 to 30% if the subdural hematoma was removed within 4 h after injury [5]. Although few reports have shown similar findings [6, 7], a number of subsequent studies have failed to identify the effect of time to surgery on mortality rate [3, 8–14]. In fact, some studies have reported a significant association between faster time to surgery and higher mortality rate [15, 16]. Thus, we evaluated the data of TASDH patients who were surgically treated from 2008 to 2015 in Chia-Yi Christian Hospital in Taiwan. In this study, the effect of time from injury to surgery (TIS) on the outcomes of TASDH patients who were in coma from the time of trauma and who did not regain consciousness before surgical intervention was examined.

## Methods

This study (CYCH-IRB 106074) was conducted after obtaining approval from the ethics committee of Chia-Yi Christian Hospital and has been performed in accordance with the ethical standards laid down in the 1964 Declaration of Helsinki and its later amendments. Patients with closed-head injury who had acute subdural hematoma on brain CT scan and who underwent craniotomy or craniectomy for the removal of hematoma were included in our study. However, patients with epidural hematoma, penetrating head injury, or intraparenchymal hemorrhage were excluded. Patients with TASDH and concomitant intraparenchymal hemorrhage that did not require evacuation were included. Between January 2008 and December 2015, a total of 235 patients from the Neurosurgical Department of Chia-Yi Christian Hospital in Taiwan met the criteria for TASDH. Patients who had thoracic, abdominal, or pelvic injury (*n* = 10) or those who did not have any record of time of injury (*n* = 6) were excluded. Based on our exclusion criteria, patients with a coma scale score > 8 (*n* = 114 patients, coma scale score of 9–15) and those aged > 70 years (*n* = 19) were not included. Furthermore, 16 patients with a coma scale score of 3 or 4 who were combined with bilateral pupil dilation were excluded, of which 13 died and three were in vegetative state. A total of 70 patients met the criteria and were included for further analysis. In this study, surgical indication and treatment of acute subdural hematoma were in accordance with the guidelines on Surgical Management of Acute Subdural Hematoma [1]. In patients who were included in the study, the following data were extracted from the medical database of our hospital: age, sex, trauma mechanism, coma scale score, pupil size, and light reflex, midline shift on brain CT scan, whether craniotomy or craniectomy and evacuation of acute subdural hematoma were performed, postoperative intracranial pressure (ICP) in the surgical intensive care unit (mean data obtained during the second day after operation), information about postoperative complications or reoperation, time of injury notification (according to ambulance station record in 56 patients or witness report in 14 patients), arrival time at the emergency room of our hospital, time of surgery initiation, and surgical outcomes. TIS was defined as the documented time of injury notification to the initiation of surgery. The surgical outcomes were assessed using the Glasgow outcome scale (GOS) at least 1 year after the injury. In most cases, the outcomes were recorded during follow-up visit to the neurosurgeon. In a few cases, the outcomes were recorded via phone call by neurosurgical staff. Functional recovery was defined as a GOS score of 4 or 5. Severe neurological deficits, vegetative state, and death were considered poor outcomes. These data was presented in detail in additional file 1.

### Statistical analysis

The student’s *t*-test,chi-square test and Mann-Whitney U test was used for the comparison of variables in the functional and poor recovery groups (Table 1). In particular, student’s *t*-test or Mann-Whitney U test was utilized to evaluate continuous or numeric variables (such as age, coma scale, TIS and midline shift on brain CT scan), and the chi-square test was used to assess non-numeric variables (including sex, traumatic mechanism, and type of operation). In addition, univariate and multivariate logistic regression models were used to analyze the impact of each variable in outcomes (Table 2). The preoperation systemic disease and postoperation comorbidities were presented in Table 3. All statistical analyses were performed using the Statistical Package for the Social Sciences software for Windows version 21.0 (IBM Corp., Armonk, NY, USA).

**Table 1:** Baseline demographic data of functional and poor recovery groups with traumatic acute subdural hematoma

		Surgical outcomes		*P*-value
		Functional recovery group	Poor recovery group	
		*n* = 37	*n* = 33	
Age (mean ± SD), years	50.9 ± 14.6	48.7 ± 13.8	53.3 ± 15.3	0.092*
	16–40	10 (27.03%)	6 (18.18%)	
	41–60	18 (48.65%)	15 (45.45%)	
	> 60	9 (24.32%)	12 (36.36%)	
Sex				0.638
	Male	25 (67.57)	24 (72.73)	
	Female	12 (32.43)	9 (27.27)	
Cause of trauma				0.729
	Traffic accidents	25 (67.57)	21 (63.64)	
	Falls and others	12 (32.43)	12 (36.36)	
Coma scale (mean ± SD)	5.9 ± 1.1	6.1 ± 1.2	5.8 ± 1.1	0.147*
	4 or 5	9 (24.32)	15 (45.45)	
	6	17 (45.95)	11 (33.33)	
	7 or 8	11 (29.73)	7 (21.21)	
Pupil size				0.306
	Normal	21 (56.76)	14 (42.42)	
	Unilateral dilation	11 (29.73)	10 (30.30)	
	Bilateral dilation	5 (13.51)	9 (27.27)	
Midline shift (mean ± SD) on brain CT scan	10.0 ± 5.2 mm	9.1 ± 4.6	10.9 ± 5.8	0.17
	< 10 mm	22 (59.46)	14 (42.42)	
	≥10 mm	15 (40.54)	19 (57.58)	
ICP				0.003
	≤25	35 (94.59)	22 (66.67)	
	> 25	2 (5.41)	11 (33.33)	
Type of surgery				0.395
	Craniectomy	21 (56.76)	22 (66.67)	
	Craniotomy	16 (43.24)	11 (33.33)	
Injury to surgery (mins)	162.5 ± 45.6	145.5 ± 27.0	181.9 ± 54.5	0.002*

*Age, coma scale and TIS are calculated with Mann-Whitney U test

**Table 2:** Univariate and multivariate regression models

	Crude OR	*P*-value	Adjusted OR	*P*-value
	(95% CI)		(95% CI)	
Age				
≤ 40	1 (reference)		1 (reference)	
41–60	1.39 (0.41–4.72)	0.598	1.36 (0.25–7.36)	0.721
> 60	2.22 (0.59–8.41)	0.240	2.89 (0.47–17.92)	0.254
Sex				
Male	1 (reference)		1 (reference)	
Female	0.78 (0.28–2.19)	0.639	0.83 (0.23–2.97)	0.779
Cause of trauma				
Traffic accidents	1 (reference)		1 (reference)	
Falls and others	1.19 (0.44–3.20)	0.730	0.85 (0.25–2.93)	0.794
Coma scale				
4 or 5	1 (reference)		1 (reference)	
6	0.39 (0.13–1.19)	0.098	0.29 (0.05–1.78)	0.489
7 or 8	0.39 (0.11–1.34)	0.133	0.34 (0.04–2.70)	0.424
Pupil reaction				
Normal	1 (reference)		1 (reference)	
Unilateral dilation	1.36 (0.46–4.06)	0.577	1.02 (0.19–5.47)	0.538
Bilateral dilation	2.70 (0.75–9.76)	0.130	2.38 (0.26–21.53	0.810
Type of surgery				
Craniectomy	1 (reference)		1 (reference)	
Craniotomy	0.66 (0.25–1.74)	0.396	0.34 (0.09–1.28)	0.111
Time (TIS, mins)	1.02 (1.01–1.04)	0.004	1.03 (1.01–1.05)	0.002

**Table 3:** Pre-operation systemic disease and post-operation comorbidity between functional and poor recovery group

		Surgical outcomes		*P*-value
		Functional recovery group	Poor recovery group	
		*n* = 37	*n* = 33	
Pre operation systemic disease	DM	7 (18.92%)	8 (24.24%)	0.77
	Cardiovascular	2 (5.41%)	0 (0.0%)	0.49
	Liver cirrhosis	1 (2.70%)	2 (6.06%)	0.59
Post operation infection	Pneumonia	12 (32.43%)	16 (48.48%)	0.22
	Urinary tract infection	3 (8.11%)	3 (9.09%)	1.0
	Brain abscess	1 (2.70%)	0 (0.0%)	1.0
Post operation seizure		8 (21.62%)	8 (24.24%)	1.0
Re-operation for intracranial hemorrhage		4 (10.81%)	4 (12.12%)	1.0

Age and coma scale were classified into three groups for analysis (Tables 1 and 2). *P*-values < 0.05 were considered statistically significant.

**Fig. 1:**
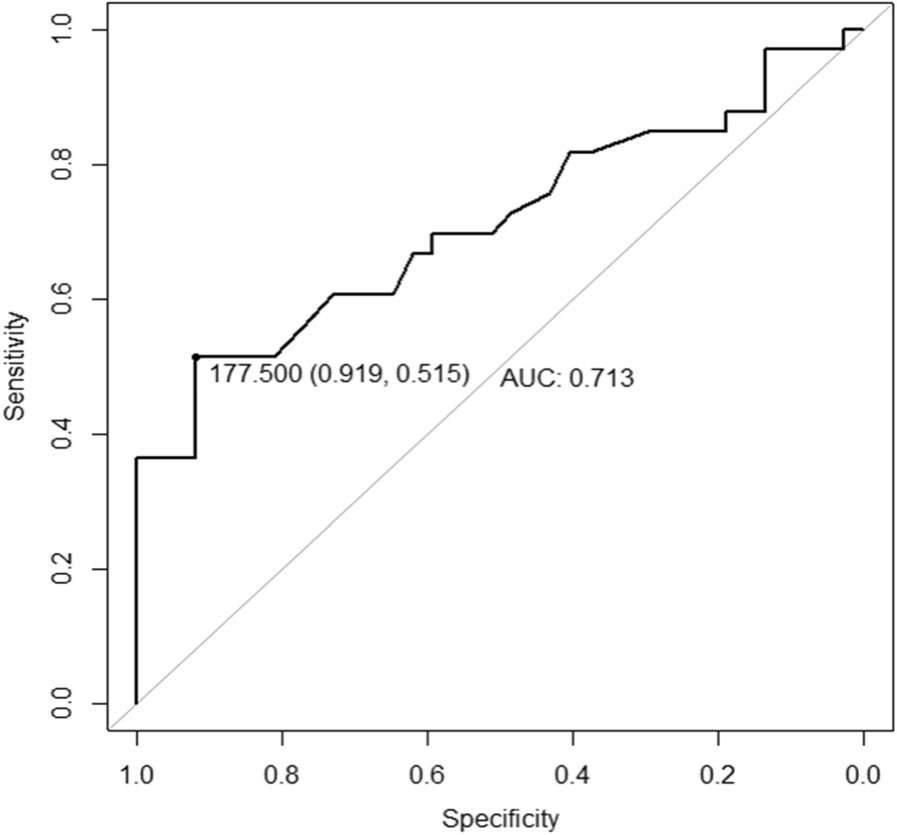
Time from injury to surgery (min) and surgical outcomes using the receiver operating characteristic curve. Area under the curve = 0.713. Threshold time: 177.50 min (2 h and 57.5 min). *P*-value = 0.002, sensitivity = 0.515, and specificity = 0.919

Finally, the receiver operating characteristic (ROC) curve was used for the analysis of TIS in the functional and poor recovery groups (Fig. 1).

## Results

Of the 235 TASDH patients who were surgically treated, 70 were included in our study. The demographic data of these patients are presented in Table 1. In total, 37 patients achieved functional recovery (*n* = 11, GOS score of 5; *n* = 26, GOS score of 4) and 33 patients had poor recovery (*n* = 15, severe neurological deficit; *n* = 9, vegetative state; *n* = 9, death). The mean age and standard deviation of the whole group was 50.9 ± 14.6 (range: 16–70) years. Among the patients, 49 (70.0%) were men and 21 (30%) women. The mean coma scale score was 5.9 ± 1.1, the mean midline shift on brain CT scan was 10.0 ± 5.2, and the mean TIS was 162.5 ± 45.6 min (Table 1).

### Difference in each variable between the functional and poor recovery groups

Age, sex, coma scale, trauma mechanism, pupil size and light reflex, type of operation (craniectomy or craniotomy), and midline shift on brain CT scan did not significantly differ between the two recovery groups (Table 1).

The postoperative ICP was significantly lower in the functional recovery group than in the poor recovery group (*p* = 0.003, *t*-test). The TIS was 145.5 ± 27.0 min in the functional recovery group and 181.9 ± 54.5 min in the poor recovery group. The Mann-Whitney U test revealed that TIS is the most significant variable in distinguishing the two recovery groups (*p* = 0.002, Table 1).

### Analysis of variables in the univariate and multivariate logistic regression models

Each variable (age, sex, coma scale, pupil size and light reflex, traumatic mechanism, type of operation and TIS) was analyzed using the univariate and multivariate logistic regression models. The results revealed that TIS was a significant factor for functional outcomes in both regression models. Age, sex, coma scale, pupil size, type of operation, and traumatic mechanism were not significantly associated with functional outcomes (Table 2).

### Significance of TIS

The TIS was analyzed with the ROC curve, and the results are shown in Fig. 1. The threshold time for functional recovery was 2 h and 57.5 min. The specificity and sensitivity were 0.919 and 0.515, respectively. This result indicated that the probability of functional recovery in a comatose TASDH patient who undergoes surgery within less than 2 h and 57.5 min was 51.5% and that of a patient who undergoes surgery after the threshold time was 8.1% (100% − 91.9%). The area under the curve was 0.713, which indicated the credibility of the ROC curve, and the *P*-value was 0.002.

### The comorbidities between the functional and poor recovery groups

Only cardiovascular disease, diabetes mellitus, or liver cirrhosis was found in preoperation systemic disease and presented in Table 3. No patients took coumadin or new oral anticoagulants before craniotomy and removal of subdural hematoma. Two patients in functional group took 100 mg aspirin because of coronary artery disease but they did not have postoperation intracranial hemorrhage. Postoperation infection, seizure, or reoperation for intracranial hematoma was also presented in Table 3. These data had no significant difference between functional and poor recovery group.

## Discussion

The present study aimed to determine whether TIS affects the degree of functional recovery in TASDH patients who required emergency craniotomy and removal of acute subdural hematoma. Various statistics were used in this study. Between the functional and poor recovery groups, only TIS showed a significant difference (Table 1). When a univariate and multivariate logistic regression model was applied, TIS had a significant effect on functional recovery (Table 2). TIS was further analyzed with the ROC curve. The threshold time for functional recovery was 2 h and 57.5 min. The specificity and sensitivity were 0.919 and 0.515, respectively. This result indicated that the probability of functional recovery in a comatose TASDH patient who undergoes surgery within less than 2 h and 57.5 min was 51.5% and that of a patient who undergoes surgery after the threshold time was 8.1% (100%–91.9%). TIS is a significant factor influencing the functional outcome in our study. In previous studies, such as that of Dent et al. (1995), counter-intuitive results have shown that a shorter TIS is correlated to poor functional recovery [15, 16]. However, such event is attributed to the existence of significant selection bias because patients with more severe injuries are more likely to undergo earlier surgery. This selection bias will certainly skew the results. Dent et al. (1995) have shown that patients who had surgery within 4 h were more likely to have a lower Glasgow coma scale score, more severe intracranial injuries, and greater incidence of brain herniation than those who had surgery after 4 h. To prevent a similar bias, only patients who had a coma scale score of 4–8, those younger than 70 years, and those who did not have additional structural brain injury other than TASDH were included. Moreover, patients with torso injuries were excluded as such conditions are commonly accompanied with hypotension and additional systemic complication. A multivariate logistic regression analysis that includes multiple variables will significantly reduce the likelihood of selection bias.

In the hypothesis of Mathai et al. (2010), the onset of life-threatening brain swelling in patients with severe TBI occurs between 2 and 3 h after the injury and may be attributed to the osmotic load exerted by the breakdown of debris in the membrane and cytoplasmic structures [17]. In the report of Haselsberger et al. (1988), the surgical outcomes of TASDH patients were influenced by preoperative consciousness status [6]. When the time interval between onset of coma and surgical decompression exceeded 2 h, the mortality rate increases from 47 to 80%. Meanwhile, Seelig et al. (1981) have reported an increase in mortality rate from 30 to 90% if TIS exceeds 4 h [5]. The current study focused on the functional outcomes of TASDH patients who were in coma and required emergency surgical operation. Our statistical analyses revealed that TIS was a significant factor and that the threshold time for surgery on TASDH patients must be assessed to achieve functional recovery.

With regard to the factors influencing outcomes, the impact of age and coma scale on functional recovery have been studied most frequently in the past [18–20]. Our data did not show that younger patients or those with a higher coma scale score were more likely to obtain better outcomes (Table 1), and this result may be attributed to two reasons. First, only 70 sets of data were included in our study, which may be considered a small sample size. Second, based on our exclusion criteria, 19 patients who were older than 70 years (*n* = 8, severe neurological deficit; *n* = 7, vegetative state; *n* = 4, dead) and 16 patients with a coma scale score of 3 or 4 combined with bilateral pupil dilatation (*n* = 3, vegetative state; *n* = 13, dead) were not included. These reasons reduced the impact of age and coma scale on outcomes in this study.

As with all studies, the present study had some limitations. It had a small sample size and was conducted at a single center. Some exclusion criteria were also applied to age and coma scale score. Nevertheless, this study can be helpful in understanding the importance of TIS in patients with TBI and can provide valuable contributions in future-related studies.

The time lapse from injury was considered a critical factor based on the study of Seelig et al. (1981) in 1981. However, several authors have obtained different conclusions [3, 9, 13, 14, 16, 17]. Our study included TASDH patients who were surgically treated from 2008 to 2015. With the use of the exclusion criteria, we believe that our sample is reasonable and that some obvious selection biases were eliminated. Thus, TIS is an important factor for the functional recovery of TASDH patients. For comatose and surgically treated TASDH patients, TIS beyond 2 h and 57.5 min would imply poor functional recovery.

## Conclusion

TIS is crucial for the functional recovery of TASDH patients who underwent surgery. The threshold time for functional recovery of comatose and surgically treated TASDH patients was 2 h and 57.5 min in our study.

## Supplementary information

**Additional file 1.**

## Data Availability

The materials described in my manuscript will be freely available to any scientist who wish to use them for non-commercial purpose. It has been presented in attached file.

## Abbreviations

CT: Computed tomography
GOS: Glasgow outcome scale
ROC: Receiver operating characteristic
TASDH: Traumatic acute subdural hematoma
TBI: Traumatic brain injury
TIS: Time from injury to surgery
DM: Diabetes mellitus
